# Sleep Quality Impairment Is Associated With Pandemic Attitudes During the Coronavirus Disease 2019 (COVID-19) Circuit Breaker Lockdown in England: A Cross-Sectional Study

**DOI:** 10.3389/fpubh.2022.819231

**Published:** 2022-07-15

**Authors:** Jonathan Kantor, Bella Nichole Kantor, Rebecca G. Fortgang, Edward F. Pace-Schott

**Affiliations:** ^1^Center for Global Health, University of Pennsylvania Perelman School of Medicine, Philadelphia, PA, United States; ^2^Center for Clinical Epidemiology and Biostatistics, University of Pennsylvania Perelman School of Medicine, Philadelphia, PA, United States; ^3^Department of Dermatology, University of Pennsylvania Perelman School of Medicine, Philadelphia, PA, United States; ^4^Florida Center for Dermatology, St Augustine, FL, United States; ^5^Department of Psychology, Harvard University, Cambridge, MA, United States; ^6^Department of Psychiatry, Harvard Medical School, Massachusetts General Hospital, Boston, MA, United States

**Keywords:** COVID-19, sleep, OPAS-C, pandemic (COVID-19), mental health

## Abstract

**Objectives:**

The COVID-19 pandemic has been associated with sleep quality impairment and psychological distress, and the general public has responded to the pandemic and quarantine requirements in a variety of ways. We aimed to investigate whether sleep quality is low during a short-term (circuit break) quarantine restriction, and whether sleep quality is associated with respondents' overall attitudes to the pandemic using a validated scale.

**Design and Setting:**

Online cross-sectional study in England in November 2020.

**Participants:**

The study included 502 respondents over the age of 18.

**Measurements:**

Sleep quality was assessed using the Pittsburgh Sleep Quality Index (PSQI), and pandemic attitudes were assessed using the Oxford Pandemic Attitudes Scale–COVID-19 (OPAS-C), a validated 20-item, 7-domain scale that assesses pandemic-related stress, fear, loneliness, sense of community, sense of exaggerated concern, non-pharmaceutical interventions, and vaccine hesitancy. Unadjusted and multivariable logistic regression odds ratios of association were assessed between the dependent variable of poor sleep quality (PSQI>5) and risk factors, including OPAS-C score, age, sex, educational status, and income.

**Results:**

The mean (SD) PSQI score was 7.62 (3.49). Overall, 68.9% of respondents met criteria for poor sleep quality using the PSQI cutoff of >5. The mean (SD) OPAS-C score was 60.3 (9.1). There was a significantly increased odds of poor sleep quality in the highest vs. lowest OPAS-C quartiles (OR 4.94, 95% CI [2.67, 9.13], *p* < 0.0001). Age, sex, income, political leaning, employment status, and education attainment were not associated with poor sleep quality.

**Conclusions:**

More than two-thirds of respondents met criteria for poor sleep quality. The odds of poor sleep quality increased in a dose-response relationship with pandemic attitudes (such as higher levels of pandemic-related stress, fear, or loneliness). The association between poor sleep quality and pandemic attitudes suggests opportunities for public health and sleep medicine interventions, and highlights the need for further research.

## Introduction

The coronavirus disease 2019 (COVID-19) pandemic has been associated with significant effects on sleep quality, and numerous studies have evaluated the intersection between the pandemic, quarantine, physical activity reduction, and mental health outcomes (1–8). Several mechanisms have been proposed for the observed impaired sleep quality associated with the COVID-19 pandemic, including increased stressors and anxiety, decreased entrainment, and decreased physical activity, and several studies have now reviewed these associations (9–12).

The psychological toll of the COVID-19 pandemic is significant, and the effect of the pandemic – modulated both through its direct effects on stress and indirect effects on schedule – has been explored for both healthcare workers and the general population (6, 13, 14). Indeed, for those with baseline psychiatric comorbidities, such problems may be even more pronounced (6). Studies have explored the sleep quality of the general public, healthcare workers, those with baseline sleep disorders, and those with baseline psychiatric comorbidities during the various phases of the COVID-19 pandemic (15–25). Several studies have also reported longitudinal data, suggested a worsening of sleep quality during the pandemic (26, 27), while others have used historical controls to assess pandemic-related sleep quality changes (28).

In November 2020, in response to an increased COVID-19 caseload and concerns regarding hospital capacity, Prime Minister Boris Johnson announced a “circuit break” quarantine would go into effect across England (29). The finite nature of this circuit break, coupled with the public's recent lived experience of 8 months of preceding restrictions, presented an opportunity to investigate the effect of limited-duration lockdowns on sleep quality. Given that sleep quality impairment has been tied to loneliness and other *chronic* stressors, whether a short-term lockdown, where the emotional stressors and overall experience is anticipated to be temporary, affects sleep quality is unknown. Since individuals may be less bothered both practically and emotionally by a temporary and finite lockdown than by restrictions that have no predetermined endpoint, and because these short-term restrictions may become a more common approach as the pandemic continues to evolve, this is an area where further research is needed.

We therefore sought to explore both whether sleep quality is low during a circuit break quarantine of finite duration and whether sleep quality is associated with respondents' overall attitudes to the pandemic using validated scales. A better understanding of these questions may have implications for both public policy and public health interventions.

## Methods

### Participants and Procedure

Participants consisted of an internet-based sample of adults residing in the UK. Inclusion criteria were age 18 years or older and current residence in the UK at the time of the study. This study was approved by the Ascension Health Institutional Review Board.

This was a cross-sectional, internet-based study conducted in November 2020. An online survey was developed using the Qualtrics platform (Qualtrics Corp, Provo, Utah) that included validated scales for sleep quality and COVID-19 attitudes, as well as other demographic questions. The survey was distributed using Prolific Academic (Oxford, United Kingdom), an established platform for academic survey research, to a database of survey respondents in the UK, and distributed using a survey panel approach (30). Respondents were rewarded with a small payment (<£1). Participants provided consent and were permitted to terminate the survey at any time. All surveys were anonymous and confidential, with linkages between data performed using a 24-character alphanumeric code. The investigators had no access to identifying information at any time.

### Sleep Quality

Sleep quality was assessed using the Pittsburgh Sleep Quality Index (PSQI), a validated 9-question scale that has been used extensively to assess sleep quality in the context of the COVID-19 pandemic (4, 22, 31–34). Scores range from 0 (no sleep quality impairment) to 21 (extreme sleep quality impairment), and a cutoff of >5 has been used since the scale's original development to define impaired sleep quality (31). Previous studies have suggested that the PSQI has a sensitivity of 89.6% and specificity of 86.5% using this cutoff for identifying impaired sleep quality (35).

### COVID-19 Attitudes

Attitudes to the COVID-19 pandemic were assessed using the Oxford Pandemic Attitudes Scale – COVID-19 (OPAS-C), a validated 20-item, 7-domain scale that assesses a range of attitudes to COVID-19 (Table 1) (36). Domains include stress, fear, loneliness, sense of community, sense of exaggerated concern, non-pharmaceutical interventions, and vaccine hesitancy. Scores range from 20 to 100, with higher values representing a greater burden and less adjustment to the pandemic.

**Table 1:** The OPAS-C.

**Number**	**Item**	**Domain**
1	I am having trouble relaxing because of the virus	Stress
2	I cannot control worrying about the virus.	Stress
3	I think about the virus more than I would like.	Stress
4	Thoughts of the virus pop into my head even when I do not want them to.	Stress
5	I have trouble concentrating because I think about the virus so much.	Stress
6	I check the news or online sources for updates on the virus more than I would like.	Stress
7	I am having trouble sleeping because I am thinking about the virus.	Stress
8	I am afraid of getting the virus myself.	Fear
9	I am afraid of a family member getting the virus.	Fear
10	I feel isolated from other people in the pandemic.	Loneliness
11	With the pandemic, I feel like I cannot connect to other people.	Loneliness
12	I feel close to other people.	Community
13	I feel part of a larger community of people.	Community
14	I think the pandemic is a hoax.	Exaggerated
15	I think people are getting too excited about the pandemic.	Exaggerated
16	I am wearing a face covering or mask when I am around people.	NPIs
17	I am social distancing.	NPIs
18	I am washing my hands frequently.	NPIs
19	I would take the coronavirus vaccine when it becomes available.	Vaccine
20	I would have my children or parents take the vaccine when it comes out.	Vaccine

*All answer choices are rated using a 5-point Likert scale (strongly disagree though strongly agree). For questions 1–13, strongly agree is scored as 5, while for questions 14–20 strongly agree is scored as a 1 (reverse scoring). Higher values reflect a greater burden, and the total score therefore ranges from 20 to 100*.

### Demographic Information

Age, sex, employment status, household income, and political affiliation were included based on self-report. Binary choices were provided for sex selection. Employment status was divided into full-time, part-time, or no employment. Income was included as a continuous variable based on total yearly household income. Political leaning was established through a Likert-style question regarding self-identification as conservative or liberal.

### Statistics

Sample size calculations were conducted for the primary endpoint of detecting a 5% difference in the OPAS-C by sleep quality status, dichotomizing between those with and without poor sleep quality using a PSQI cutoff of 5. 442 subjects (221 per group) would be adequate to detect a 5% change in OPAS-C with 80% power and with an alpha of 0.05, assuming a baseline OPAS-C mean of 56.1 with a standard deviation of 10.5 and assuming equal group sizes (37).

Demographic data are presented as mean values with 95% confidence intervals (CI). *T*-tests and chi-squared tests were used as appropriate for continuous and categorical variables, respectively. Unadjusted and multivariable logistic regression odds ratios of association were assessed between the dependent variable of poor sleep quality (defined as PSQI>5) and putative risk factors, including OPAS-C score, age, sex, educational status, and income. Respondents were also divided into quartiles based on OPAS-C score, and both mean PSQI values and the proportion meeting poor sleep quality criteria were presented by quartiles; the significance of interquartile differences was assessed using analysis of variance.

All statistical analyses were performed using Stata 13 for Mac (Stata Corporation, College Station, Texas).

## Results

### Characteristics

Of the 513 subjects who were recruited, 502 completed the survey, yielding a completion rate of 97.9%. All completed surveys were received during the lockdown. The mean (SD) age of respondents was 34.2 (12.8), and 341 (69.5%) of the respondents were female; respondent characteristics are outlined in Table 2. Demographic data did not differ significantly between those that did and did not meet criteria for poor sleep quality.

**Table 2:** Demographic and characteristics of respondents, overall and by sleep quality status.

**Characteristic**	**No. (%)**		
		Poor Sleep Quality*	
	**Total**	**Yes**	**No**
Overall	502 (100)	346 (68.9)	156 (31.1)
Sex			
Men	150 (30.6)	97 (46.1)	53 (54.3)
Women	341 (69.5)	241 (53.9)	100 (45.7)
Age, y			
18–30	244 (48.6)	171 (70.1)	73 (29.9)
31–40	121 (24.1)	80 (66.1)	41 (33.9)
41–50	69 (13.8)	52 (75.4)	17 (24.6)
51–60	44 (8.8)	28 (63.6)	16 (36.40
>60	24 (4.8)	15 (62.5)	9 (37.5)
Education level			
< High school	1 (0.2)	0 (0)	1 (100)
High school	131 (26.2)	96 (73.3)	35 (26.7)
Some college	101 (20.2)	71 (70.3)	30 (29.7)
Bachelor's	184 (36.7)	119 (64.7)	65 (35.3)
Graduate	84 (16.8)	59 (70.2)	25 (29.8)
Employment status			
Full time	201 (46.6)	141 (70.2)	60 (29.9)
Part time	102 (23.7)	70 (68.6)	32 (31.4)
Not employed	128 (29.7)	87 (68.0)	41 (32.0)
Income			
<£10,000	57 (11.4)	41 (71.9)	16 (28.1)
£10,000–£30,000	171 (34.1)	119 (69.6)	52 (30.4)
£30,001–£50,000	147 (29.3)	105 (71.4)	42 (28.6)
£50,001–£80,000	80 (15.9)	50 (62.5)	30 (37.5)
£80,001–£100,000	20 (4.0)	12 (60.0)	8 (40.0)
£>100,000	27 (5.4)	19 (70.4)	8 (29.6)
Political leaning			
Conservative	81 (16.1)	55 (67.9)	26 (32.1)
Liberal	261 (52.0)	183 (70.1)	78 (29.9)
Ambivalent	160 (31.9)	108 (67.5)	52 (32.5)

* *Defined as PSQI>5*.

### Sleep Quality

The mean (SD) PSQI score was 7.62 (3.49) with a range of 1-20. Overall, 68.9% (*n* = 346) of respondents met criteria for poor sleep quality using the established PSQI cutoff of >5.

### Pandemic Attitudes

The mean (SD) OPAS-C score was 60.3 (9.1), with a range of 38 to 80; for reference, the mean (SD) OPAS-C score in the UK assessed in July 2020 during the original OPAS-C validation study was 56.1 (10.5) (36). The mean (SD) OPAS-C subscale scores were as follows: stress 19.9 (6.9); fear 7.8 (1.9); loneliness 10.5 (3.2); sense of community 5.3 (2.0); concern that the pandemic is exaggerated 8.3 (1.8); attitude to non-pharmaceutical interventions (NPIs) 4.4 (1.9); and attitude to vaccination 4.2 (2.5). The OPAS-C subscales have not been separately validated, and no cutoffs have been established for a negative or dysfunctional attitude to the COVID-19 pandemic.

### Association Between Sleep Quality and Pandemic Attitudes

Stratifying overall PSQI scores and the proportion meeting poor sleep quality criteria by OPAS-C quartiles demonstrated a progressive worsening of sleep quality as pandemic attitudes worsened, as seen in Table 3 (*p* < 0.0001). Unadjusted logistic regression analysis demonstrated that OPAS-C scores were significantly associated with poor sleep quality (OR 1.07, 95% CI [1.05, 1.10], *p* < 0.0001 for each unit increase in OPAS-C score). There was a significantly increased odds of poor sleep quality in the highest versus lowest OPAS-C quartiles (OR 4.94, 95% CI [2.67, 9.13], *p* < 0.0001), suggesting that poor sleep quality is associated with less positive or healthy pandemic attitudes. These associations persisted in fully adjusted models (OR 1.07, 95% CI [1.05, 1.10], *p* < 0.0001 for each unit increase in OPAS-C score and OR 4.88, 95% CI [2.51, 9.48], *p* < 0.0001 for the highest versus lowest OPAS-C quartiles). In a secondary analysis, association of poor sleep quality with the individual OPAS-C subscales varied by subscale (Table 4).

**Table 3:** Sleep quality stratified by OPAS-C quartile.

**OPAS-C Quartile**	**Mean (95% confidence intervals)**	
	**PSQI raw score**	Proportion of respondents meeting cutoff criteria for poor sleep quality*
Quartile 1	6.45 (5.93, 6.96)	0.54 (0.46, 0.63)
Quartile 2	7.08 (6.43, 7.73)	0.59 (0.50, 0.68)
Quartile 3	7.80 (7.28, 8.33)	0.78 (0.71, 0.86)
Quartile 4	9.35 (8.66, 10.04)	0.85 (0.79, 0.92)

* *Defined as PSQI>5*.

**Table 4:** Association of individual OPAS-C subscale scores with the likelihood of being a poor sleeper (defined as PSQI>5).

**OPAS-C Subscale**	**Odds of being a poor sleeper (per 1-point increase in each subscale)**	***P*-value**
Stress	1.11 (1.07, 1.14)	<0.0001
Fear	1.16 (1.05, 1.28)	0.003
Loneliness	1.16 (1.09, 1.23)	<0.0001
Community	0.82 (0.75, 0.91)	<0.0001
Exaggerated	1.01 (0.91, 1.13)	0.787
NPIs	0.86 (0.78, 0.95)	0.002
Vaccine	1.02 (0.95, 1.11)	0.560

Age (OR 0.99, 95% CI [0.98, 1.01], *p* = 0.421), sex (OR 0.76, 95% CI [0.51, 1.14], *p* = 0.186 for male sex), income (OR 0.93, 95% CI [0.80, 1.08], *p* = 0.352), political leaning (OR 0.98, 95% CI [0.55, 1.74], *p* = 0.950), employment status (OR 0.90, 95% CI [0.56, 1.46], *p* = 0.676 for unemployed vs. full time), and educational attainment (OR 1.08, 95% CI [0.65, 1.80], *p* = 0.765 for those with a graduate degree vs. all others) were not significantly associated with sleep quality on logistic regression analyses with categorical variables. A fully adjusted logistic regression model similarly did not demonstrate any significant associations.

## Discussion

We found that the November 2020 circuit break was associated with impaired sleep quality in the UK, and that the degree of sleep quality impairment was associated with pandemic attitudes as assessed with the OPAS-C. Every 1-point increase in the OPAS-C score – with higher scores representing worsening attitudes to the pandemic –was associated with a 7.4% increase in odds of being a poor sleeper. Thus for each 1-SD increase in OPAS-C score, the odds of being a poor sleeper increased by 66.7%. An important strength of this study was our use of validated scales for assessing both sleep quality and pandemic attitudes.

Both the raw PSQI scores and the proportion of respondents meeting criteria for poor sleepers appeared high when compared with historical controls, though without a longitudinal design it is impossible to determine this definitively. The mean PSQI score in an Italian general population prior to the COVID-19 pandemic was 4.0 and approximately 35% of the population met criteria for poor sleep, though one study of young adults in Spain suggested a pre-pandemic mean PSQI of 5.8 with 47% meeting criteria for poor sleep pre-pandemic (38–40).

We did not detect an association between poor sleep quality and several demographic variables, such as age, sex, employment status, political leaning, and income. While the study may have been underpowered to detect these associations, this also bolsters the effect size of our finding that pandemic attitudes are associated with poor sleep quality. A prior study evaluated the relationship between sleep quality and pandemic attitudes, and also found an association between impaired sleep quality and dysfunctional pandemic attitudes, though it did not use a validated scale for pandemic attitudes and focused exclusively on worry, stress, and adverse life impact related to COVID-19 (41).

Several studies have demonstrated that with a shift to lockdown, where the majority of the population is restricted from working and leaving their homes on a regular basis, the absence of an early morning awakening drive may lead to both a reduction in social jetlag, as weekdays and weekends functionally merge, and a more delayed chronotype (42). Thus, the decreased entrainment seen as part of the loss of zeitgebers may be responsible for some of the sleep onset delay seen in the pandemic context (43, 44). In addition to delayed onset, a modest increase in sleep quantity has been observed in several studies (45).

Several other mechanisms may be responsible for the effect of the COVID-19 pandemic on sleep, and these were not directly evaluated in this study; some have suggested that decreased physical activity brought on by quarantine leads to sleep impairment and ensuing poor mental health outcomes, and decreased physical activity itself may be associated directly with the poor mental health outcomes as well (46, 47). Sleep impairment may also be associated with an increase in electronic device usage and other sedentary behavior, further exacerbating this feedback loop (39). Moreover, decreased daylight exposure due to activity restrictions may lead to a further reduction in entrainment induced by the primary zeitgeber (48, 49). Finally, dietary changes are another possible contributor to pandemic-related sleep impairment, as this may also affect both sleep itself as well as the likelihood of engaging further in physical activity (5).

Given the association between sleep disorders and mental health outcomes, and the potential effects of both pandemic-related stress and reduced entrainment on sleep, the COVID-19 pandemic may represent a perfect storm, as unhealthy behaviors such as decreased activity couple with decreased daylight exposure, reduced work-related zeitgebers, and general stress induced by both schedule change and pandemic-related fears to produce a sleep-unfriendly environment. Thus the combination of stress and reduced entrainment may be partly responsible for a decrease in sleep quality during the pandemic (50). Given the social responsibility for sleep researchers to educate the general public and healthcare providers regarding sleep in the pandemic context, further highlighting the importance of research investigating the intersection between COVID-19 and sleep quality is of significant value (48).

Loneliness and perceived social support may represent important considerations when attempting to understand the intersection between sleep and the COVID-19 pandemic. Loneliness may be responsible for part of the sleep quality impairment seen in older adults, and this may combine with an increased baseline prevalence of sleep quality disturbance to result in an elevated risk of poor pandemic-context sleep in older adults (51). Moreover, one study demonstrated a dose-response relationship between social support and sleep quality, and a similar modulating effect between social support and mental health outcomes such as depression and anxiety (52). Furthermore, self-esteem may modulate the effects of stress on both anxiety and sleep (9), and false beliefs may also affect sleep quality (53), while habituation may lead to a gradual improvement in sleep quality (54), further complicating the psychological constructs underlying sleep impairment. Finally, impaired sleep may interact further with underlying psychological processes and result in impaired immune function, with potentially serious effects in a pandemic context (55, 56).

As seen in Table 4, there was a variable association of individual OPAS-C subscale scores with poor sleep quality, with the stress, fear, and loneliness subscales associated with worse sleep quality and community and NPI subscales associated with improved sleep quality. While stress, fear, and loneliness are known to be associated with impaired sleep quality, the sense of community and NPI subscales of the OPAS-C increase for those who are less concerned with the effects of the pandemic—and thus are associated with decreased stress—potentially explaining their protective association with sleep quality. Indeed, these findings echo work that has suggested that media consumption regarding the pandemic is associated with more severe symptoms of depression (57).

Despite evidence regarding the negative sleep quality effects of the COVID-19 pandemic, some evidence, particularly from early in the pandemic course, suggested that the net effect on sleep quality was salutary, so that most healthy adults were sleeping more – and better – than before the pandemic (41). Still, even in that study those most vulnerable to sleep impairment before the onset of the pandemic were most likely to experience sleep quality decline in the pandemic context (41).

Our study has several limitations. First, the generalizability of our findings may be limited by the non-representative nature of our population. This is a particularly important problem given the potential interaction between type of work, risk of COVID-19 exposure, and sleep quality (1). Second, as with any survey study, response bias and social desirability bias may affect the validity of the data, though the anonymous survey design may help mitigate these concerns. Third, our selection of independent variables was not exhaustive, and other important variables, such as family stress (58), underlying mental health diseases (59), and others may be important confounders. Fourth, the composite OPAS-C score is heterogeneous, capturing a range of attitudes on disparate pandemic responses such as fear and vaccination concerns; future studies validating the component subscale scores for use independently, and evaluating the ideal ways in which the composite scores should be used, would be beneficial. Finally, this cross-sectional study lacks a comparator group and cannot establish causation; therefore, we do not know whether the associations we describe truly represent clinical risk factors. Future prospectively designed studies evaluating outcomes over several longitudinal timepoints with representative populations may be helpful.

Both impaired sleep quality and pandemic attitudes – including a tendency to eschew non-pharmaceutical interventions and vaccination – may be associated with an increased risk of COVID-19 infection or worsening long-term outcomes (56, 60). Therefore, the public health implications of these findings raise the specter of a synergistic interaction between poor sleep, COVID-19 attitudes, decision-making and ultimate outcomes. Sleep quality during the limited-duration circuit-break quarantine in the UK was impaired, and poor sleep was strongly associated with less desirable attitudes to the pandemic. The dose-response relationship between impaired sleep quality and pandemic attitudes has important implications for further research and suggests potential avenues for possible sleep quality and public health interventions in the future.

## Data Availability Statement

The raw data supporting the conclusions of this article will be made available by the authors, without undue reservation.

## Ethics Statement

The studies involving human participants were reviewed and approved by Ascension Health IRB. The patients/participants provided their written informed consent to participate in this study.

## Author Contributions

JK: study conception. JK and BK: data collection. EP-S, RF, and BK: critical editorial review. EP-S and RF: supervision. All authors contributed to the article and approved the submitted version.

## Conflict of Interest

The authors declare that the research was conducted in the absence of any commercial or financial relationships that could be construed as a potential conflict of interest.

## Publisher's Note

All claims expressed in this article are solely those of the authors and do not necessarily represent those of their affiliated organizations, or those of the publisher, the editors and the reviewers. Any product that may be evaluated in this article, or claim that may be made by its manufacturer, is not guaranteed or endorsed by the publisher.
